# Vertebral artery dissection due to an esophageal foreign body migration: a case report

**DOI:** 10.11604/pamj.2014.17.96.3443

**Published:** 2014-02-07

**Authors:** Najib Benmansour, Naouar Ouattassi, Amine Benmlih, Mohamed Noureddine Elalami

**Affiliations:** 1 ENT Head and Neck Surgery Department, Hassan II University Hospital, Fez, Morocco; 2 Sidi Mohammed Benabdellah University, Fez, Morocco

**Keywords:** Vertebral artery, dissection, foreign body, Surgery

## Abstract

Unintentional foreign bodies‘ swallowing is a fairly common occurrence in ENT consultation especially among children. They usually pass through the gastrointestinal tract without complications. Migration of a foreign body through the esophageal wall is rare. It represents about 1% to 4% of all cases of foreign bodies‘ ingestion. A 16 year's old female patient has presented to ENT emergency with a painful dysphagia following an accidental ingestion of a metallic pin. Cervical X ray confirmed the presence of the pin while endoscopic investigations have shown no foreign body. Cervical CT scan revealed the migration of the foreign body through the esophageal wall with left vertebral artery dissection. Endoscopic management has been sufficient with an uneventful post operative follow up. Esophageal foreign bodies are very diverse mainly dominated by fish bones (60%) and chicken bones (16%). Metallic pins are rare. The major risks of migration of those foreign bodies are cervical abscess, mediastinitis and oeso-vascular fistulae. Cases of self extrusion through the skin have been reported. Migration of a foreign body through the esophageal wall is rare. Endoscopic management has been sufficient.

## Introduction

Accidental foreign bodies ‘ swallowing is frequent in daily ENT practice. Usually it is an unthreatening incident and the foreign body is easily found and removed at ENT investigation either by simple examination or by rigid endoscopy under general anesthesia or it pass through the gastrointestinal tract without complication. Esophageal wall penetration and migration of foreign bodies is uncommon. Its incidence is reported to be between 1% and 4% [1]. It could be a life threatening condition as the foreign body could cause serious cervical, mediastinal suppuration or vascular complications. Vascular complications due to esophageal foreign bodies ‘ migration are very unusual. In fact, over 588 publications on PubMed about complications of foreign bodies ‘ migration, only six are related to vascular complications that were mainly represented by eso-vascular fistulae. We believe this is the first case of vascular dissection due to an esophageal foreign body migration.

## Patient and observation

**Figure 1:**
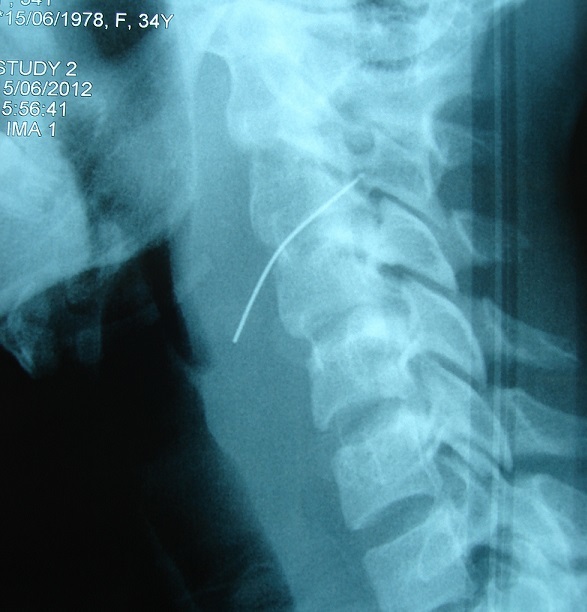
Lateral neck X ray showing a metallic pin at the level of the 3rd and 4th cervical vertebrae

**Figure 2:**
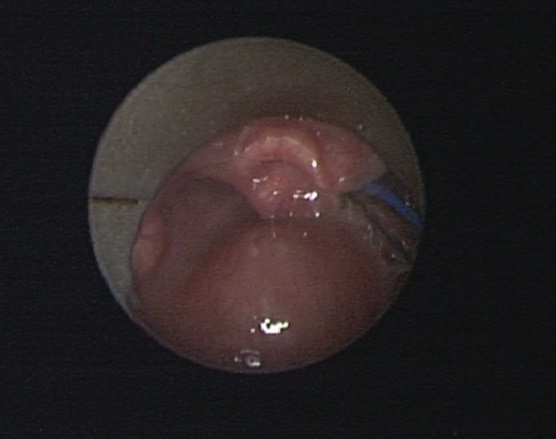
Endoscopic view showing no foreign body

**Figure 3:**
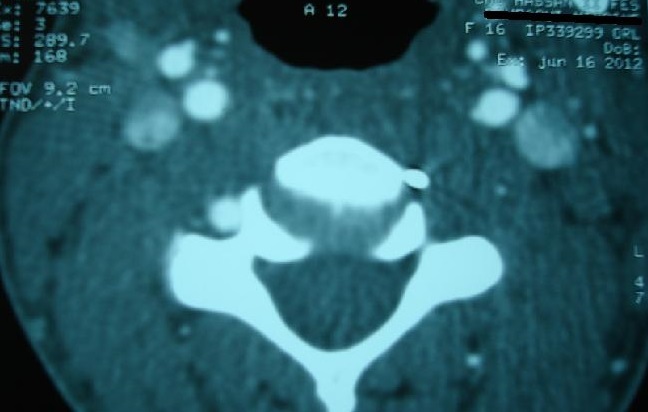
CT scan axial view showing a metallic pin beside the left transverse foramen of the third cervical vertebrae

**Figure 4:**
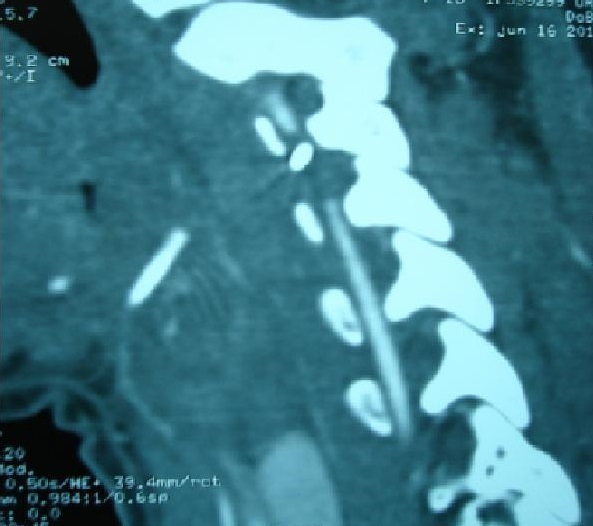
CT scan para sagittal view showing a metallic pin that passes through the transverse foramen of the third cervical vertebrae with interruption of the vertebral artery

**Figure 5:**
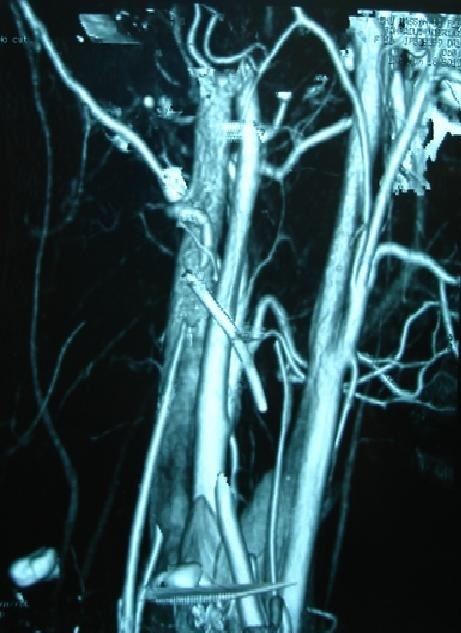
Cervical CT angiography that shows a metallic pin with interruption of blood flow within the left vertebral artery

A sixteen year's old female patient has presented to ENT emergency with a painful dysphagia following an accidental ingestion of a metallic pin without haematemesis or chest pain or dyspnea. The patient was apyretic with stable hemodynamic and no evidence of subcutaneous emphysema on physical examination. Cervical X ray confirmed the presence of the metallic pin (Figure 1) while endoscopic investigations have shown no foreign body (Figure 2). It found only a bulging of the left wall of the hypopharynx with no bruising or mucosal wound. Cervical CT scan (Figure 3, Figure 4, Figure 5) revealed the migration of the foreign body through the esophageal wall and the left transverse foramen of the third cervical vertebrae with left vertebral artery dissection.

The metallic pin was successfully found under hypopharyngoscopy and esophagoscopy with the operating fluoroscopy guidance. After mucosal incision performed on the hypopharynx left wall bulging, the pin has been removed with a forceps clamp. A nasogastric tube was set up for a week. Antibiotherapy was maintained for 10 days and antiplatelet therapy for 3 months. The patient was discharged from the hospital after 3 days. Post operative follow up has been uneventful over 10 months.

## Discussion

In litterature the majority of foreign bodies ingested become impacted in the tonsils, base of tongue or vallecula and can be easily removed in the clinic. In few cases the foreign body becomes lodged at one of the constrictions along the esophagus, requiring removal by rigid oesophagoscopy under general anesthesia. In even fewer cases, the foreign body penetrates the esophageal mucosa and “migrates” through it. In some instances, the foreign body can migrate completely through the esophageal wall and become impacted in the soft tissues of the neck [2–4]. A migrated foreign body can remain silent or cause serious suppurating or vascular complications [5]. The most frequent ingested foreign bodies in the Ear Nose and Throat sphere are chicken and fish bones [6]. In our practice, many female patients presented to our emergency department with metallic foreign bodies such as scarf-pins or needles which were held in their mouth while they were adjusting their scarves or dress-making. The symptoms are immediate and patients quickly seek for medical help. If not found at ENT examination, standards X ray can help diagnosing radio-opaque foreign bodies. Lateral neck X rays though useful, do not help determine if migration has occurred. Foreign body migration is suspected on the basis of suggestive history, a positive finding on lateral neck radiography, and a negative finding on rigid oesophagoscopie [7]. CT scan helps defining the location of the migrating foreign body and diagnosing complications and though planning the therapeutic strategy. Main complications of foreign-body migration include retropharyngeal abscess, perforation of the esophagus, perforation of the aorta, embedment in the thyroid gland, and migration through the common carotid artery [7, 8]. When such a case presents, most authors [7–9] agree that management involves exploration of the neck by an external approach to identify the foreign body and remove it after performing an injected cervical CT scan that help planning the surgical procedure. However in those cases the foreign body was organic and couldn ‘t be located otherwise. A case has been reported in 1958 of a foreign body (needle) migrating into the common carotid artery, the foreign body was extracted directly by endoscopy [10]. In our case, fluoroscopy has been of a great help in locating the foreign body.

In litterature the majority of foreign bodies ingested become impacted in the tonsils, base of tongue or vallecula and can be easily removed in the clinic. In few cases the foreign body becomes lodged at one of the constrictions along the esophagus, requiring removal by rigid oesophagoscopy under general anesthesia. In even fewer cases, the foreign body penetrates the esophageal mucosa and “migrates" through it. In some instances, the foreign body can migrate completely through the esophageal wall and become impacted in the soft tissues of the neck [2–4]. A migrated foreign body can remain silent or cause serious suppurating or vascular complications [5]. The most frequent ingested foreign bodies in the Ear Nose and Throat sphere are chicken and fish bones [6]. In our practice, many female patients presented to our emergency department with metallic foreign bodies such as scarf-pins or needles which were held in their mouth while they were adjusting their scarves or dress-making. The symptoms are immediate and patients quickly seek for medical help. If not found at ENT examination, standards X ray can help diagnosing radio-opaque foreign bodies. Lateral neck X rays though useful, do not help determine if migration has occurred. Foreign body migration is suspected on the basis of suggestive history, a positive finding on lateral neck radiography, and a negative finding on rigid oesophagoscopie [7]. CT scan helps defining the location of the migrating foreign body and diagnosing complications and though planning the therapeutic strategy. Main complications of foreign-body migration include retropharyngeal abscess, perforation of the esophagus, perforation of the aorta, embedment in the thyroid gland, and migration through the common carotid artery [7, 8]. When such a case presents, most authors [7–9] agree that management involves exploration of the neck by an external approach to identify the foreign body and remove it after performing an injected cervical CT scan that help planning the surgical procedure. However in those cases the foreign body was organic and couldn ‘t be located otherwise. A case has been reported in 1958 of a foreign body (needle) migrating into the common carotid artery, the foreign body was extracted directly by endoscopy [10]. In our case, fluoroscopy has been of a great help in locating the foreign body.

## Conclusion

Serious complications following foreign body ingestion though rare can be life threatening. They include stricture formation, intramural abscess and formation of fistula tracts. Management has to be as quick as possible in a qualified medical institution. CT scan is fundamental to assess the extent of damage and plan the management strategy that could be through an external approach or an endoscopic investigation.
